# Secondhand smoke, genetic susceptibility, and incident chronic kidney disease in never smokers: A prospective study of a selected population from the UK Biobank

**DOI:** 10.18332/tid/162607

**Published:** 2023-05-10

**Authors:** Rui Lan, Xue Li, Xiangjun Chen, Jinbo Hu, Wenjin Luo, Liangjing Lv, Yan Shen, Yao Qin, Lina Mao, Hanwen Ye, Qifu Li, Zhihong Wang

**Affiliations:** 1 Department of Endocrinology, The First Affiliated Hospital of Chongqing Medical University, Chongqing, China

**Keywords:** secondhand smoke, genetic susceptibility, chronic kidney disease, UK Biobank

## Abstract

**INTRODUCTION:**

A large number of people around the world are exposed to the risks of passive smoking. This prospective study aimed to examine the association between secondhand smoke exposure, exposure time, and the incidence of chronic kidney disease (CKD) and determine whether this association was influenced by genetic susceptibility.

**METHODS:**

The study included 214244 participants of the UK Biobank who were initially free of CKD. Cox proportional hazards model was used to estimate the associations between secondhand smoke exposure time and the risks of CKD in people who have never smoked. The genetic risk score for CKD was calculated by a weighted method. The likelihood ratio test comparing models was used to examine the cross-product term between secondhand smoke exposure and genetic susceptibility to CKD outcomes.

**RESULTS:**

During a median of 11.9 years of follow-up, 6583 incidents of CKD were documented. Secondhand smoke exposure increased the risk of CKD (HR=1.09; 95% CI: 1.03–1.16, p<0.01), and a dose-response relationship between CKD prevalence and secondhand smoke exposure time was found (p for trend<0.01). Secondhand smoke exposure increases the risk of CKD even in people who never smoke and have a low genetic risk (HR=1.13; 95% CI: 1.02–1.26, p=0.02). There was no statistically significant interaction between secondhand smoke exposure and genetic susceptibility to CKD (p for interaction=0.80).

**CONCLUSIONS:**

Secondhand smoke exposure is associated with higher risk of CKD, even in people with low genetic risk, and the relationship is dose dependent. These findings change the belief that people with low genetic susceptibility and without direct participation in smoking activities are not prone to CKD, emphasizing the need to avoid the harm of secondhand smoke in public places.

## INTRODUCTION

Chronic kidney disease (CKD) is a global economic and health burden. In 2017, the prevalence of CKD was 9.1% (range: 8.5–9.8) and is increasing every year^1,2^. CKD is associated with serious health problems, hospitalization, cardiovascular disease, mortality, etc.^3-5^. While CKD is an irreversible chronic disease, early identification of risk factors for CKD could effectively prevent the occurrence or delay the development of CKD^6^.

Smoking is a modifiable lifestyle factor that receives considerable public attention among the traditional risk factors for CKD. Smoking can be divided into active smoking and passive smoking. Although most countries have implemented national smoke-free workplace legislation to reduce harm from secondhand smoke exposure, the rate of secondhand smoke exposure in people remains high^7-11^. Most studies to date have focused on the association between active smoking and the risk of CKD^12^; consequently, the relationship between passive smoking and CKD remains unclear. One study enrolled 1948 participants and investigated the risk of CKD in adults who never smoked but were exposed to secondhand smoke^13^. The study found that secondhand smoke exposure >3 days per week increased the risk of CKD; however, exposure time was not evaluated in the study. The impact of secondhand smoke exposure time on the risk of CKD needs to be further elucidated. In addition, it is widely accepted that genetic and behavioral factors contribute to the development of CKD, but whether secondhand smoke exposure modifies the effect of genetic predisposition on CKD remains largely unknown^14-16^.

This study utilized UK Biobank prospective data to: 1) determine the relationship between secondhand smoke exposure, exposure time and CKD; and 2) examine the joint association of secondhand smoke exposure and genetic susceptibility with CKD outcomes and explore potential gene–secondhand smoke interactions.

## METHODS

### Study population

**Figure 1:**
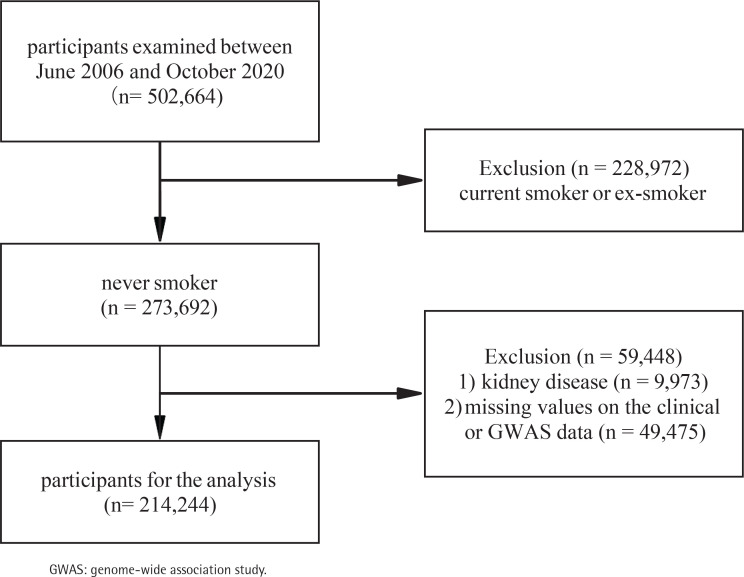
Flow diagram of selection of study population from the UK Biobank

The UK Biobank^17^ is a large-scale biomedical database containing genetic and health information from 502664 participants aged 40–69 years in the UK between 2006 and 2010; details of the study design and methods have been described elsewhere previously^16,18^. The current study analysis was approved by UK Biobank (ID 66536). The analysis received approval from the National Information Governance Board for Health and Social Care and the National Health Service North West Multicentre Research Ethics Committee (UK) (reference 13/NW/0382). All participants had provided informed consent through electronic signature at the first assessment. At baseline, participants who were current smokers or ex-smokers (n=228972), or participants with CKD (n=9973), or missing values on the clinical or genome-wide association study (GWAS) data (n=49475) were excluded, leaving 214244 participants for the analysis (Figure 1).

### Assessment of secondhand smoke exposure

Passive exposure to secondhand smoke was based on self-reporting. Participants were asked to report their smoking status, and response options were ‘never’, ‘previous’, ‘current’, ‘prefer not to answer’, and ‘don’t know’. Participants who answered ‘never’ were further asked: ‘At home, about how many hours per week are you exposed to other people’s tobacco smoke?’, ‘At outside home, about how many hours per week are you exposed to other people’s tobacco smoke?’. Based on the answers to those questions, the participants’ current secondhand smoke exposure status and exposure time were derived.

### Study outcome

Chronic kidney diseases were classified based on the International Classification of Diseases (10th Edition), including diabetes mellitus with renal complications (E10.2, E11.2, E12.2, E13.2, and E14.2), hypertensive renal disease (I12 and I13), glomerular disease (N03, N04, N05, and N07), renal tubulointerstitial disease (N11, N12, N13, N14, and N15), and renal failure (N18 and N19)^19^. These data were recorded in the UK Biobank study through hospital admissions, self-reported information, or death registration.

### Definition of genetic risk score (GRS)

UK Biobank genotyping, quality control, and imputation procedures are run by the UK Biobank professional team and have previously been described in detail^20^. A total of 241 independent single nucleotide polymorphisms (SNPs) were selected, which were identified from the most recent GWAS and were significantly associated with CKD; detailed information on the selected SNPs is provided in Supplementary Table 1^21^. The GRS for CKD was calculated by a method that has been described elsewhere:


$$GRS\;=\;\left(\beta_{1}\times SNP_{1}\;+\;\beta_{2}\times SNP_{2}\;+\;\ldots \;+\;\beta_{241}\times SNP_{241}\right)$$

with each SNP coded 0, 1, or 2, according to the number of risk alleles. The *β* coefficient was obtained from the reported GWAS meta-analysis^21^. Genetic risk scores are divided into three quartiles for high, medium, and low genetic risk.

### Assessment of other covariates

Demographic and lifestyle behaviors were collected via a touchscreen device. The Townsend deprivation index (based on the participant’s postcode; higher scores indicate a higher degree of deprivation) was obtained. Physical activity was reflected by total metabolic equivalent task (MET) minutes per week for all activity and divided into three quantiles as low, medium, and high. Education level classification was based on the presence or absence of a college education. Diet was defined as healthy or unhealthy according to the AHA guidelines^22^. Blood and urine samples were collected and analyzed in the central laboratory.

### Statistical analysis

All analyses were performed using R v4.1.2 (http://www.R-project.org, The R 121 Foundation). The follow-up time was calculated from recruitment date to date of diagnosis of outcome or last follow-up date, whichever came first. Baseline characteristics were analyzed according to whether participants were exposed to secondhand smoke, with categorical variables presented as frequency and percentage, and normally distributed continuous variables as mean ± standard deviation (SD). Cox proportional hazards model was used to calculate the relationship between secondhand smoke exposure and CKD outcome and estimate the hazard ratio (HR) and 95% confidence interval (CI). We screened for confounding factors that were closely related to CKD using previous literature and clinical findings, and tried to avoid statistical collinear factors. Multivariate models were established for controlling potential confounders, including age, sex, race, alcohol consumption, physical activity, Townsend deprivation index, body mass index, total triglyceride, diabetes mellitus, hypertension, and CKD-GRS. To check the reliability of the results, adjustments were also made for additional social factors like education level and healthy diet. The likelihood ratio test comparing models was used to examine whether there was a cross-product term between secondhand smoke exposure and genetic susceptibility to CKD outcomes. A p<0.05 represents statistical significance.

## RESULTS

The baseline characteristics of 214244 participants were grouped according to whether they were exposed to secondhand smoke. Compared with participants who were not exposed to secondhand smoke, those with secondhand smoke exposure tended to be female, younger, more physically active, had lighter alcohol consumption, higher baseline eGFR levels, and a higher BMI. Participants with secondhand smoke exposure also appeared to be more materially deprived and likely to have diabetes mellitus, cardiovascular disease, high cholesterol, and hypertension (Table 1).

**Table 1:** Baseline characteristics of selected study population from the UK Biobank (N=214244)

*Characteristics*	*Secondhand smoke*			*p*
	*Total (N=214244) n (%)*	*No exposure (N=170433) n (%)*	*Exposure (N=4381) n (%)*	
**Gender** (Female)	90246 (42.1)	68685 (40.3)	21561 (49.2)	<0.01
**Age** (years), mean (SD)	68.3 (8.1)	68.6 (8.1)	67.5 (8.1)	<0.01
**Race** (White)	196354 (91.6)	157316 (92.3)	39038 (89.1)	<0.01
**Alcohol intake frequency**				<0.01
Daily or almost daily	33893 (15.8)	27285 (16.0)	6608 (15.1)	
3–4 times a week	50052 (23.4)	40174 (23.6)	9878 (22.5)	
1–2 times a week	59744 (27.9)	47364 (27.8)	12380 (28.3)	
1–3 times a month	26397 (12.3)	20860 (12.2)	5537 (12.6)	
Special occasions only	25623 (12.0)	20179 (11.8)	5444 (12.4)	
Never	18535 (8.7)	14571 (8.5)	3964 (9.0)	
**Physical activity**				0.79
Low	50888 (23.8)	40460 (23.7)	10428 (23.8)	
Medium	61063 (28.5)	48634 (28.5)	12429 (28.4)	
High	102293 (47.7)	81339 (47.7)	20954 (47.8)	
**Health status**				
TDI, mean (SD)	-1.7 (2.9)	-1.9 (2.7)	-0.9 (3.2)	<0.01
DM	14211 (6.6)	10255 (6.0)	3956 (9.0)	<0.01
CVD	10008 (4.7)	7475 (4.4)	2533 (5.8)	<0.01
Hypertension	51321 (24.0)	39326 (23.1)	11995 (27.4)	<0.01
***Laboratory data***	***Mean (SD)***	***Mean (SD)***	***Mean (SD)***	
eGFR (mL/min/1.73 m^2^)	93.6 (15.7)	93.5 (15.6)	93.9 (15.9)	<0.01
BMI (kg/m^2^)	27.0 (4.7)	26.8 (4.6)	28.0 (5.0)	<0.01
SBP (mmHg)	137.0 (18.3)	137.0 (18.4)	136.9 (18.0)	0.38
DBP (mmHg)	82.2 (10.0)	82.0 (10.0)	82.8 (10.2)	<0.01
FBG (mmol/L)	5.1 (1.1)	5.1 (1.1)	5.1 (1.3)	<0.01
TC (mmol/L)	5.7 (1.1)	5.7 (1.1)	5.7 (1.1)	<0.01
TG (mmol/L)	1.7 (1.0)	1.6 (0.9)	1.7 (1.0)	<0.01
HDL-c (mmol/L)	1.5 (0.4)	1.5 (0.4)	1.4 (0.4)	<0.01
LDL-c (mmol/L)	3.6 (0.8)	3.6 (0.8)	3.6 (0.9)	<0.01
HbA1c (%)	5.4 (0.6)	5.4 (0.5)	5.4 (0.6)	<0.01

DM: diabetes mellitus. CVD: cardiovascular disease. TDI: Townsend deprivation index. SBP: systolic blood pressure. DBP: diastolic blood pressure. BMI: body mass index. WC: waist circumference. FPG: fasting plasma glucose. HbA1c: glycated hemoglobin. TG: total triglyceride. TC: total cholesterol. HDL-c: high density lipoprotein cholesterol. LDL-c: low density lipoprotein cholesterol. eGFR: estimated glomerular filtration rate.

During the study, with a median of 11.9 years of follow-up, 6583 incidents of CKD were recorded. The median duration of passive smoking to CKD was 9.18 years. Cox proportional hazards model was used to calculate the relationship between secondhand smoke exposure and CKD outcome. In Model 1, which was adjusted for age, race, and sex, secondhand smoke exposure increased the risk of CKD in never smokers by 25% (HR=1.25; 95% CI: 1.18–1.32, p<0.01). In Model 2, further adjustments were made for alcohol consumption, physical activity, and Townsend deprivation index, and secondhand smoke exposure was associated with a HR of 1.19 (95% CI: 1.13–1.26, p<0.01) for CKD. Model 3 was additionally adjusted for diabetes mellitus, hypertension, total triglyceride, body mass index, and CKD-GRS, and an increased risk of kidney disease in never smokers was still observed with secondhand smoke exposure (HR=1.09; 95% CI: 1.03–1.16, p<0.01) (Table 2).

**Table 2:** Multivariable adjusted HRs (95% CIs) for CKD by secondhand smoke among the study population within a 11.9-year follow-up (N=214244)

*Secondhand smoke*	*Model 1*	*Model 2*	*Model 3*
	*HR (95% CI)*	*HR (95% CI)*	*HR (95% CI)*
No exposure (Ref.)	1	1	1
Exposure	1.25 (1.18–1.32)	1.19 (1.13–1.2)	1.09 (1.03–1.16)

Model 1: adjusted for age, sex, and race. Model 2: adjusted as in model 1 + physical activity, Townsend deprivation index, and alcohol consumption. Model 3: as in model 2 + total triglyceride, body mass index, hypertension, diabetes mellitus, and CKD-GRS.

To increase the reliability of the results, sensitivity analyses were performed. Following further adjustment of social influencing factors (education level, healthy diet), the associations were slightly attenuated but remained significant (HR=1.08; 95% CI: 1.02–1.14, p<0.01), as presented in Supplementary file Table 2. Considering the reverse causal association, the participants who developed CKD within the first 2 years of follow-up were further excluded, and the results were similar to those in Model 3 (HR=1.09; 95% CI: 1.03–1.15, p<0.01, table not shown).

Subsequently, secondhand smoke exposure time was quantified, and after multivariable adjustment, exposure time ≤3 h and >3 h increased the risk of CKD compared with no exposure; adjusted HR was 1.07 (95% CI: 1.01–1.14, p<0.01) and 1.14 (95% CI: 1.02–1.27, p=0.02) for exposure time ≤3 h and >3 h, respectively. Furthermore, secondhand smoke exposure time exhibited a significant dose-response relationship with the risk of CKD (p for trend <0.01) (Table 3). The absolute risk of developing CKD within 5 years was 94% for the study participants who were exposed to secondhand smoke for >3 hours per week and 87% for those who were exposed to secondhand smoke for ≤3 hours per week; these were increases of 13% and 6%, respectively, compared with the participants who were not exposed to secondhand smoke (81%).

**Table 3:** Multivariable adjusted HRs (95% CIs) for CKD by the quantile of exposure time among the study population within a 11.9-year follow-up (N=214244)

*Secondhand smoke*	*Model 1*	*Model 2*	*Model 3*
	*HR (95% CI)*	*HR (95% CI)*	*HR (95% CI)*
No exposure (Ref.)	1	1	1
Exposure time ≤3 h	1.19 (1.11–1.26)	1.15 (1.08–1.23)	1.07 (1.01–1.14)
p	<0.01	<0.01	<0.01
Exposure time >3 h	1.50 (1.35–1.67)	1.35 (1.21–1.50)	1.14 (1.02–1.27)
p	<0.01	0.03	0.02
p for trend	<0.01	<0.01	<0.01

Model 1: adjusted for age, sex, and race. Model 2: adjusted as in model 1 + physical activity, Townsend deprivation index, and alcohol consumption. Model 3: as in model 2 + total triglyceride, body mass index, hypertension, diabetes mellitus, and CKD-GRS.

**Figure 2:**
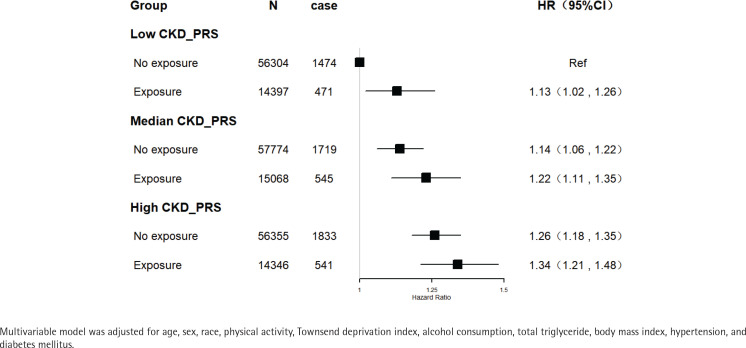
The joint association of genetic risk and secondhand smoke with CKD among the study population within a 11.9-year follow-up (N=214244)

Polygenic risk scores for CKD were also calculated. Secondhand smoke exposure was associated with increased risks of CKD among participants with low, medium, or high genetic risk. Compared with people with low genetic risk and no exposure to secondhand smoke, those exposed to secondhand smoke had increased risk of CKD even in those who never smoked and had a low genetic risk (HR=1.13; 95% CI: 1.02–1.26, p=0.02). Participants with a high genetic risk of CKD and exposure to secondhand smoke had a 34% increased risk of incident CKD (HR=1.34; 95% CI: 1.21–1.48, p<0.01) (Figure 2). However, there was no statistically significant interaction between secondhand smoke exposure and genetic susceptibility to CKD (p for interaction=0.80).

## DISCUSSION

This large prospective population-based study revealed that secondhand smoke exposure increases the risk of CKD and that the secondhand smoke exposure time has a significant dose-response relationship with CKD. Moreover, to our knowledge, this is the first study reporting the joint association of secondhand smoke exposure and polygenetic risk score of CKD with the CKD outcomes.

The relationship between secondhand smoke and CKD has been explored in a few studies. The cross-sectional study of Omoloja et al.^23^ evaluated the secondhand smoke exposure status of children through questionnaires and urine nicotine content, and showed that secondhand smoke exposure was independently associated with proteinuria in children (OR=2.64; 95% CI: 1.08–6.42). Another cross-sectional study in the United States included 7516 adolescents aged 12–17 years for the determination of serum creatinine and cotinine, among which 3692 adolescents were exposed to secondhand smoke, and the results showed that adolescents exposed to secondhand smoke had lower eGFR compared with those not exposed to secondhand smoke (mean difference= -2.2, 95% CI: -4.0 – -0.4)^24^. Only a limited number of prospective studies have examined the association between secondhand smoke exposure and the risk of CKD. Our study extends the results of the study of Jhee et al.^13^ which comprised a longitudinal sub-cohort of 1948 participants and found that a frequency of secondhand smoke exposure <3 days/week was not associated with the risk of CKD compared with those who were not exposed to secondhand smoke (HR=1.58; 95% CI: 0.94–2.66), but a secondhand smoke exposure frequency >3 days/week was associated with the risk of CKD (HR=1.62; 95% CI: 1.03–2.63)^13^. In our study, the relationship between exposure time and incidence of CKD was further investigated and secondhand smoke exposure was found to be associated with the risk of incidence of CKD, while significant dose-response relationships between the exposure time and CKD were also observed. Dose-response relationships between secondhand smoke exposure and major tobacco-related mortality were found in previous secondhand smoke exposure cohort studies such as that of He et al.^25^. The dose-dependent association between active smoking and CKD has long been documented in studies related to kidney disease^26,27^. Thus, the various studies collectively show that passive and active smoking have similar effects on kidney disease.

Many people around the world are caught in the myth that they are not directly involved in harmful behavior or do not have a genetic predisposition or family history of a disease and therefore they are not susceptible to illness. However, the polygenic risk scores for CKD that were calculated in this study indicate that secondhand smoke exposure increases the risk of CKD, even in people who never smoke and have a low genetic risk. This underscores the necessity of protecting population health by enhancing smoking bans in public places and homes.

Several potential mechanisms could explain the observed associations between passive smoking and the increased risk of CKD. Animal studies suggest that passive smoking can not only lead to kidney and vascular fibrosis by increasing the myeloperoxidase reaction, but can also increase lipid peroxidation, change renal morphology and structure, and damage renal function^28-32^. There is also evidence that secondhand smoke exposure leads to the accumulation of cytotoxic substances^33^.

### Strengths and limitations

This study strengths include that it is the first to explore the relationship between secondhand smoke exposure time and the risk of developing CKD, which complements existing studies on the relationship between secondhand smoke and CKD. Also, this is the first study to combine secondhand smoke exposure and genetic susceptibility to CKD outcomes. Although no significant interaction between secondhand smoke exposure and genetic susceptibility was observed, ameliorating the environmental factors of secondhand smoke exposure may offset at least some of the high genetic risk of CKD.

The study does have some limitations. First, secondhand smoke exposure time was obtained through self-reporting instead of more objective methods, such as direct measurement of serum nicotine content. Despite the inevitable subjective error of recall bias, a questionnaire is more convenient, easy to popularize, and a large sample size can be obtained. Second, the data on secondhand smoke exposure were only collected at baseline, and considering the limitations of observational studies, the causal relationship between secondhand smoke exposure and the risk of CKD cannot be determined. Third, although the multi-factor corrected Cox regression method used for the study and the results are acceptable and credible, unmeasured or unknown residual confounding is inevitable.

## CONCLUSIONS

Secondhand smoke exposure was associated with CKD among people who never smoke and have a low genetic risk. Since secondhand smoke exposure is a modifiable harmful factor, this study highlights the need to protect against CKD harm from secondhand smoke exposure. Furthermore, the study provides a theoretical basis for primary prevention of CKD.

## Data Availability

The data supporting this research are available from the authors on reasonable request.

## References

[1] GBD Chronic Kidney Disease Collaboration (2020). Global, regional, and national burden of chronic kidney disease, 1990-2017: a systematic analysis for the Global Burden of Disease Study 2017. Lancet..

[2] Eckardt KU, Coresh J, Devuyst O (2013). Evolving importance of kidney disease: from subspecialty to global health burden. Lancet..

[3] Kelly DM, Ademi Z, Doehner W (2021). Chronic kidney disease and cerebrovascular disease: consensus and guidance from a KDIGO controversies conference. Stroke..

[4] Go AS, Chertow GM, Fan D, McCulloch CE, Hsu CY (2004). Chronic kidney disease and the risks of death, cardiovascular events, and hospitalization. N Engl J Med..

[5] Toyoda K, Ninomiya T (2014). Stroke and cerebrovascular diseases in patients with chronic kidney disease. Lancet Neurol..

[6] Romagnani P, Remuzzi G, Glassock R (2017). Chronic kidney disease. Nat Rev Dis Primers..

[7] Öberg M, Jaakkola MS, Woodward A, Peruga A, Prüss-Ustün A (2011). Worldwide burden of disease from exposure to second-hand smoke: a retrospective analysis of data from 192 countries. Lancet..

[8] Burki TK (2021). WHO releases latest report on the global tobacco epidemic. Lancet Oncol..

[9] Verma M, Kathirvel S, Das M, Aggarwal R, Goel S (2020). Trends and patterns of second-hand smoke exposure amongst the non-smokers in India- A secondary data analysis from the Global Adult Tobacco Survey (GATS) I & II. PLoS One..

[10] Xi B, Liang Y, Liu Y (2016). Tobacco use and second-hand smoke exposure in young adolescents aged 12-15 years: data from 68 low-income and middle-income countries. Lancet Glob Health..

[11] Zeng J, Yang S, Wu L (2016). Prevalence of passive smoking in the community population aged 15 years and older in China: a systematic review and meta-analysis. BMJ Open..

[12] Xia J, Wang L, Ma Z (2017). Cigarette smoking and chronic kidney disease in the general population: a systematic review and meta-analysis of prospective cohort studies. Nephrol Dial Transplant..

[13] Jhee JH, Joo YS, Kee YK (2019). Secondhand smoke and CKD. Clin J Am Soc Nephrol..

[14] Fan M, Sun D, Zhou T (2020). Sleep patterns, genetic susceptibility, and incident cardiovascular disease: a prospective study of 385 292 UK biobank participants. Eur Heart J..

[15] Li X, Zhou T, Ma H (2021). Healthy sleep patterns and risk of incident arrhythmias. J Am Coll Cardiol..

[16] Maidstone RJ, Turner J, Vetter C (2021). Night shift work is associated with an increased risk of asthma. Thorax..

[17] Bycroft C, Freeman C, Petkova D (2018). The UK Biobank resource with deep phenotyping and genomic data. Nature..

[18] Vetter C, Dashti HS, Lane JM (2018). Night shift work, genetic risk, and type 2 diabetes in the UK Biobank. Diabetes Care..

[19] Si J, Yu C, Guo Y (2018). Chronic hepatitis B virus infection and risk of chronic kidney disease: a population-based prospective cohort study of 0.5 million Chinese adults. BMC Med..

[20] Kramer I, Hooning MJ, Mavaddat N (2020). Breast cancer polygenic risk score and contralateral breast cancer risk. Am J Hum Genet..

[21] Wuttke M, Li Y, Li M (2019). A catalog of genetic loci associated with kidney function from analyses of a million individuals. Nat Genet..

[22] Lloyd-Jones DM, Hong Y, Labarthe D (2010). Defining and setting national goals for cardiovascular health promotion and disease reduction: the American Heart Association’s strategic Impact Goal through 2020 and beyond. Circulation..

[23] Omoloja A, Jerry-Fluker J, Ng DK (2013). Secondhand smoke exposure is associated with proteinuria in children with chronic kidney disease. Pediatr Nephrol..

[24] García-Esquinas E, Loeffler LF, Weaver VM, Fadrowski JJ, Navas-Acien A (2013). Kidney function and tobacco smoke exposure in US adolescents. Pediatrics..

[25] He Y, Jiang B, Li LS (2012). Secondhand smoke exposure predicted COPD and other tobacco-related mortality in a 17-year cohort study in China. Chest..

[26] Hall ME, Wang W, Okhomina V (2016). Cigarette smoking and chronic kidney disease in African Americans in the Jackson Heart Study. J Am Heart Assoc..

[27] Jin A, Koh WP, Chow KY, Yuan JM, Jafar TH (2013). Smoking and risk of kidney failure in the Singapore Chinese health study. PLoS One..

[28] Omoloja A, Tyc VL (2015). Tobacco and the pediatric chronic kidney disease population. Pediatr Nephrol..

[29] Kim DH, Suh YS, Mun KC (2004). Tissue levels of malondialdehyde after passive smoke exposure of rats for a 24-week period. Nicotine Tob Res..

[30] Boor P, Casper S, Celec P (2009). Renal, vascular and cardiac fibrosis in rats exposed to passive smoking and industrial dust fibre amosite. J Cell Mol Med..

[31] Moraes CA, Breda-Stella M, Carvalho CAF (2021). Morphofunctional study on the effects of passive smoking in kidneys of rats. Einstein (Sao Paulo)..

[32] Dündar M, Kocak I, Culhaci N (2004). Effects of long-term passive smoking on the diameter of glomeruli in rats: histopathological evaluation. Nephrology (Carlton)..

[33] Biswas S, Gairola CG, Das SK (2002). Passive cigarette smoke and the renal glyoxalase system. Mol Cell Biochem..

