# Supplementary material for: Secondhand smoke, genetic susceptibility, and incident chronic kidney disease in never smokers: A prospective study of a selected population from the UK Biobank
Source: Tob Induc Dis. 2023 May 10;21:58. doi: 10.18332/tid/162607 (PMC10170651; doi:10.18332/tid/162607)
Supplement: Supplementary file 1 [file TID-21-58-s1.pdf]

Supplementary Files

Secondhand smoke, genetic susceptibility, and incident chronic kidney disease in never smokers: a prospective study of 214,244 UK Biobank participants.

Table of contents

Supplementary Table 1. Components of the genetic risk score by study.

Supplementary Table 2. Further adjustment for the sociodemographic HRs (95% CIs) for CKD by secondhand smoke among 185,929 participants CKD within a 11.9-year follow-up.

Supplemental File Table 1. Components of the genetic risk score by study.

SNP	CHR	BP	A1	A2	OR	P
rs116626164	1	50975227	A	T	1.1	0.0028
rs116760613	1	51490709	A	G	1.09	0.0022
rs72661397	1	52013915	T	C	0.93	0.0083
rs760077	1	155178782	A	T	1.02	0.016
rs2383531	1	186729401	A	G	0.93	4.30E-05
rs3791760	2	10118424	T	C	0.98	0.013
rs11123170	2	113978940	C	G	0.97	1.10E-05
rs1078442	2	121988924	A	C	1.04	2.70E-06
rs72929920	2	177097022	T	C	1.14	7.50E-07
rs1047891	2	211540507	A	C	1.05	3.00E-08
rs2332036	3	121714391	T	C	1.02	0.0035
rs28817415	4	77401452	T	C	1.07	1.90E-16
rs12509595	4	81182554	T	C	1.07	5.10E-14
rs1229984	4	100239319	T	C	1.05	0.0025
rs11745300	5	34504668	C	G	0.96	3.10E-06
rs4976646	5	176788570	T	C	0.95	2.40E-10
rs7766720	6	107172979	T	C	0.97	0.0097
rs162185	6	134226147	T	C	0.98	0.019
rs13230625	7	1286244	A	G	1.07	8.50E-13
rs700753	7	46753684	C	G	0.97	0.0011
rs55914958	7	101237753	T	C	0.98	0.037
rs73728279	7	151411494	T	G	1.11	7.00E-24
rs7834797	8	23759535	A	G	1.04	1.20E-06

rs7084402	10	60265404	A	G	1.02	0.025
rs10821944	10	63785089	T	G	0.98	0.01
rs7096822	10	126664166	T	C	1.02	0.011
rs3925584	11	30760335	T	C	1.08	2.70E-21
rs11039216	11	47406592	T	C	1.04	2.10E-05
rs7123489	11	65524252	A	C	1.06	1.60E-11
rs4567493	11	86634423	A	G	1.02	0.014
rs7931938	11	111207105	A	G	1.03	0.0019
rs7936300	11	122601034	A	G	0.98	0.045
rs2122982	12	57781893	A	G	0.96	5.40E-05
rs1275609	12	76271183	A	G	0.97	0.0012
rs584480	13	72345505	T	C	0.98	0.04
rs7327286	13	73713447	A	G	0.97	0.0038
rs17730281	15	53907948	A	G	0.92	1.40E-18
rs4886755	15	76298132	A	G	0.97	2.60E-05
rs12908437	15	99287375	T	C	1.02	0.008
rs77924615	16	20392332	A	G	0.81	6.70E-86
rs8050136	16	53816275	A	C	1.03	2.80E-05
rs6504021	17	59240473	T	C	1.04	2.60E-05
rs16942713	18	24386535	T	G	1.07	1.00E-09
rs8096658	18	77156537	C	G	0.94	2.00E-12
rs7259714	19	817394	T	C	0.98	0.0093
rs838144	19	49250239	T	C	0.97	0.0027
rs6026580	20	57468150	T	C	0.98	0.018
rs2823139	21	16576783	A	G	1.05	1.20E-08
rs74748843	1	10730910	T	C	1.06	0.0037
rs12061708	1	18809916	A	G	1.03	0.00074
rs2749153	1	23699340	A	G	1.04	5.00E-06
rs688540	1	48002447	A	G	1.04	0.007
rs17413465	1	55718708	A	C	0.98	0.04
rs1757915	1	56615809	A	G	0.97	8.50E-05
rs679843	1	78707493	T	C	0.98	0.039
rs11166440	1	100808363	A	G	0.98	0.0045
rs10857788	1	110012289	A	G	0.98	0.0078
rs12736457	1	113258293	C	G	0.94	2.30E-06
rs267738	1	150940625	T	G	1.05	2.10E-05
rs4971100	1	155155731	A	G	0.98	0.049
rs3845534	1	163738950	A	G	1.03	0.00049
rs4656220	1	170649277	T	C	0.98	0.013
rs3850625	1	201016296	A	G	0.97	0.024
rs2808454	1	207231751	A	T	0.97	5.20E-05
rs75625374	1	208039431	C	G	0.95	0.0047
rs417237	1	228532195	T	G	0.98	0.0017
rs2490391	1	243469669	A	C	1.07	3.70E-17

rs3791221	2	226933	A	G	0.98	0.0076
rs807624	2	15782471	T	G	0.98	0.0055
rs4491726	2	18676276	A	G	0.98	0.022
rs780093	2	27742603	T	C	0.97	1.50E-05
rs2301343	2	40680149	T	G	1.02	0.044
rs2971880	2	54885640	A	T	1.04	5.00E-06
rs6546869	2	73895765	A	G	0.94	6.70E-08
rs11123169	2	113967075	T	C	0.95	2.30E-10
rs11694902	2	121988884	A	G	0.95	2.00E-04
rs7425436	2	148759656	A	G	0.98	0.044
rs4664475	2	152387553	T	C	1.03	0.0017
rs35472707	2	169995581	T	C	1.09	1.60E-05
rs187355703	2	176993583	C	G	0.82	2.80E-10
rs35284526	2	178121524	A	C	0.97	0.0017
rs4666821	2	183077254	T	G	0.97	0.00088
rs60980181	2	188168567	A	T	1.03	0.0015
rs1548945	2	217665788	T	C	0.96	1.70E-06
rs1050816	2	220358198	T	C	0.97	0.00016
rs13003198	2	234257105	T	C	0.98	0.0067
rs795009	3	12208671	T	G	0.97	0.0024
rs6778731	3	13947504	T	C	1.02	0.01
rs6779998	3	30749965	A	G	1.03	8.20E-05
rs11914389	3	38527215	T	C	0.98	0.0026
rs7651407	3	48443816	T	C	0.97	0.001
rs4625	3	49572140	A	G	1.02	0.032
rs3774726	3	63974477	T	C	1.03	4.30E-05
rs2289746	3	105455955	T	C	1.02	0.046
rs9868185	3	121657593	A	G	0.97	0.00074
rs10934754	3	125906237	T	C	0.97	0.00016
rs7624084	3	141093285	T	C	0.97	1.60E-05
rs1397764	3	141750810	A	G	0.97	9.00E-04
rs56065557	3	185354216	C	G	1.02	0.015
rs11919484	3	186432839	T	G	1.03	4.60E-05
rs9823161	3	193811168	A	G	0.98	0.0097
rs16874073	4	23743962	T	C	1.08	3.90E-05
rs4864890	4	52686513	T	C	1.02	0.022
rs223471	4	103698786	C	G	0.98	0.0022
rs71606723	4	115498457	A	T	0.97	0.0033
rs13159523	5	676962	A	G	1.03	0.00029
rs13157326	5	34504277	A	G	1.04	2.60E-06
rs1362800	5	39378115	T	C	1.06	4.60E-11
rs495237	5	39950266	T	G	0.97	0.00061
rs11746506	5	44812566	T	C	0.98	0.0062
rs12520984	5	52787358	C	G	0.98	0.015

rs79760705	5	53298716	T	G	0.95	0.00091
rs72759880	5	67750213	T	G	1.05	0.00039
rs2010352	5	68656327	A	G	1.02	0.01
rs12163971	5	132226669	A	C	1.04	0.00098
rs11743174	5	148524820	T	C	0.97	0.00068
rs3812036	5	176813404	T	C	1.07	1.20E-12
rs3765502	6	24354045	T	C	0.98	0.03
rs144100226	6	34180297	T	C	0.92	0.00036
rs13200335	6	41690823	A	C	0.98	0.0046
rs77915916	6	43287722	A	T	0.95	0.00077
rs881858	6	43806609	A	G	1.06	6.40E-11
rs720989	6	44765535	T	G	0.97	0.00029
rs12212034	6	51492862	T	C	1.02	0.0049
rs6458868	6	52630153	T	C	1.02	0.0048
rs3925003	6	55422618	T	C	1.03	3.70E-05
rs72912510	6	90118764	A	G	1.05	2.00E-05
rs1857859	6	100894587	A	G	0.98	0.005
rs7740107	6	130374461	A	T	0.95	1.30E-06
rs9375818	6	131882078	A	G	1.04	2.20E-06
rs3822939	6	133849789	A	G	1.03	0.00017
rs12207180	6	160633107	A	T	1.09	1.10E-10
rs62435145	7	1286567	T	G	1.07	2.90E-12
rs6968554	7	17287106	A	G	1.02	0.014
rs3750081	7	32930876	T	G	1.03	0.00029
rs55773927	7	65337902	T	C	0.98	0.015
rs801193	7	66030612	T	G	1.02	0.047
rs41301394	7	75612803	T	C	0.97	0.00014
rs6973656	7	77422583	A	G	0.98	0.0028
rs62491533	7	129564134	T	C	1.03	0.0031
rs10254101	7	151415536	T	C	1.11	1.20E-25
rs12671694	7	155665959	T	C	0.97	0.00085
rs868822	7	156252939	T	G	0.95	2.60E-08
rs2980423	8	8142575	T	C	1.03	0.002
rs1533059	8	8684953	A	G	0.98	0.01
rs35353426	8	9297246	T	C	1.03	0.0023
rs7832708	8	10190040	T	C	0.98	0.015
rs11783418	8	10841858	A	G	1.02	0.036
rs10098664	8	11417493	T	C	1.02	0.018
rs34861762	8	23748420	T	C	1.04	1.20E-07
rs10102889	8	32435620	C	G	1.04	0.005
rs2976178	8	87332552	C	G	1.03	0.00036
rs2954017	8	126476873	T	C	0.98	0.047
rs13287724	9	33169034	A	T	1.05	0.00055
rs544169	9	33956791	A	G	0.98	0.0094

rs2039424	9	71432174	A	G	0.96	5.40E-08
rs1321917	9	119324929	C	G	1.02	0.037
rs7024579	9	139100413	T	C	0.98	0.034
rs80282103	10	899071	A	T	0.91	9.90E-11
rs6481598	10	29781798	C	G	0.96	3.50E-05
rs7072591	10	35150364	A	G	0.98	0.024
rs10821905	10	52646093	A	G	0.97	0.0073
rs7475348	10	69965177	T	C	0.96	7.20E-06
rs12240572	10	75016365	A	T	1.04	0.0025
rs816850	10	79252446	C	G	1.02	0.04
rs7095954	10	82209232	A	T	1.02	0.0085
rs2068888	10	94839642	A	G	1.02	0.0019
rs4918943	10	97278922	A	G	1.03	0.00045
rs284859	10	104573017	T	G	0.97	0.0078
rs1536225	10	105202318	T	G	1.02	0.014
rs1055256	10	126446592	A	G	0.97	2.00E-04
rs11564722	11	2178330	T	C	0.98	0.031
rs63934	11	2789062	A	G	0.97	0.002
rs963837	11	30749090	T	C	1.08	1.60E-21
rs6484504	11	31424823	T	C	1.03	0.00068
rs61897431	11	47427667	T	C	0.96	3.20E-07
rs7127946	11	48250675	T	C	0.96	6.20E-08
rs2727040	11	49057603	T	C	1.06	7.80E-07
rs1813937	11	50468801	T	C	0.95	1.30E-07
rs948493	11	65552154	T	C	1.06	7.50E-12
rs11237450	11	78023356	A	C	0.96	3.80E-05
rs6589750	11	119326726	A	G	0.97	0.0014
rs10790452	11	121584931	T	C	0.96	3.10E-05
rs632887	12	3392351	A	G	0.97	0.0011
rs4238020	12	4616642	T	C	0.96	0.0035
rs117113238	12	12209203	A	G	0.96	0.021
rs10846157	12	15325031	A	C	1.05	2.10E-06
rs2634675	12	48740855	A	G	0.97	0.0013
rs12313306	12	57751854	T	C	0.96	5.30E-05
rs41284816	13	50655989	T	G	1.09	0.0083
rs6574652	14	81870100	T	C	1.02	0.019
rs1028455	14	88829975	A	T	0.97	0.00076
rs61993680	14	100752644	A	C	1.02	0.012
rs12913015	15	39305443	T	C	0.96	3.30E-06
rs6492982	15	41399951	T	C	1.03	0.00036
rs1145077	15	45683795	T	G	1.07	2.50E-16
rs690428	15	53950578	A	C	1.07	9.80E-15
rs956006	15	62808539	T	C	0.98	0.0078
rs11071738	15	63580155	T	C	1.03	0.00014

rs351237	15	74477239	A	G	1.02	0.021
rs4886696	15	75664570	A	T	1.02	0.02
rs438339	16	2003425	T	C	0.96	0.029
rs1635404	16	3747042	T	G	1.04	0.00016
rs9932625	16	51735746	A	G	1.03	0.00012
rs7185391	16	68323115	T	G	1.03	0.00084
rs62050038	16	69802865	A	T	0.96	0.00031
rs62053077	16	71643669	T	G	1.03	0.0016
rs28581385	16	79942679	A	T	1.03	0.0098
rs28735420	17	12139964	T	G	0.95	0.00061
rs9891340	17	17543846	T	C	0.98	0.0029
rs2440165	17	19428719	T	C	0.95	2.70E-07
rs2411192	17	34882998	A	T	1.05	4.20E-09
rs4794813	17	37670994	A	T	0.96	7.60E-05
rs227731	17	54773238	T	G	0.97	0.00053
rs9903801	17	58915261	C	G	0.97	0.0013
rs9895661	17	59456589	T	C	0.95	4.70E-07
rs8866	17	65373979	C	G	1.02	0.031
rs1719934	18	5585158	A	G	0.96	4.20E-06
rs16942751	18	24393213	A	C	1.08	2.10E-10
rs4940525	18	59354616	T	C	0.98	0.0068
rs2974751	19	13053034	A	C	0.98	0.013
rs8101667	19	33402419	T	C	0.96	1.70E-06
rs7251730	19	36997147	T	C	0.98	0.048
rs78241494	19	37649748	T	C	1.03	0.0084
rs113445505	19	38157969	T	C	0.97	0.0033
rs281380	19	49214470	T	C	1.03	0.00034
rs34647824	19	50138143	A	C	1.04	3.30E-05
rs1041606	20	14677788	T	C	1.02	0.042
rs6087579	20	32985155	A	G	1.03	0.0013
rs2273684	20	33529766	T	G	0.98	0.0022
rs17216707	20	52732362	T	C	1.05	1.30E-05
rs2235826	20	56143169	A	T	1.03	0.0013
rs1407040	20	57472174	T	C	0.98	0.027
rs35636653	20	60858758	T	C	0.97	0.00034
rs72629024	20	62152519	C	G	0.96	0.0016
rs4408777	20	62706105	A	G	1.04	2.30E-06
rs2834317	21	35356706	A	G	1.05	0.00028
rs131263	22	30133045	T	C	0.97	0.0041
rs80576	22	36539804	A	G	1.04	0.00053
rs4820324	22	38599857	C	G	1.03	0.001
rs738527	22	43112961	T	C	0.96	3.80E-05

SNP: single nucleotide polymorphism; CHR Chromosome;

Supplemental File Table 2. Further adjustment for the sociodemographic HRs (95% CIs) for CKD by secondhand smoke among 185,929 participants within a 11.9-year follow-up.

Secondhand Smoke	model3<br>HR(95% CI)	model4<br>HR(95% CI)
No exposure	1(reference)	1(reference)
Exposure	1.09（1.03 1.16）	1.08（1.01 1.14）

Model3: age(continuous), sex (male/female), ethnic (paleface/other), physical activity(low/median/high), townsend deprivation index (continuous), alcohol consumption (frequence1-6), total triglyceride(continuous), body mass index (continuous), hypertension (yes/no), diabetes mellitus(yes/no), CKD-GRS(continuous).

Model4：model3+education(yes/no)+ health diet(yes/no).

© 2023 Lan R. et al.
